# Characterization of French Coriander Oil as Source of Petroselinic Acid

**DOI:** 10.3390/molecules21091202

**Published:** 2016-09-08

**Authors:** Evelien Uitterhaegen, Klicia A. Sampaio, Elisabeth I. P. Delbeke, Wim De Greyt, Muriel Cerny, Philippe Evon, Othmane Merah, Thierry Talou, Christian V. Stevens

**Affiliations:** 1 Laboratoire de Chimie Agro-Industrielle, Université de Toulouse, INP, ENSIACET, 4 Allée Emile Monso, BP 44362, 31030 Toulouse Cedex 4, France; Evelien.Uitterhaegen@ensiacet.fr (E.U.); Muriel.Cerny@ensiacet.fr (M.C.); Philippe.Evon@ensiacet.fr (P.E.); Othmane.Merah@ensiacet.fr (O.M.); Thierry.Talou@ensiacet.fr (T.T.); 2 Laboratoire de Chimie Agro-Industrielle, Institut National de la Recherche Agronomique, 31030 Toulouse Cedex 4, France; 3 SynBioC, Department of Sustainable Organic Chemistry and Technology, Ghent University, Coupure Links 653, B-9000 Ghent, Belgium; Klicia_Sampaio@yahoo.com.br (K.A.S.); Elisabeth.Delbeke@UGent.be (E.I.P.D.); 4 EXTRAE, Food Engineering Department, University of Campinas, Rua Monteiro Lobato, 80, 13083-862 Campinas-São Paulo, Brazil; 5 Desmet-Ballestra Group, Corporate Village, Da Vincilaan 2, 1935 Zaventem, Belgium; Wim.De.Greyt@desmetballestra.com

**Keywords:** *Coriandrum sativum* L., vegetable oil, petroselinic acid, tocols, phytosterols

## Abstract

Coriander vegetable oil was extracted from fruits of French origin in a 23% yield. The oil was of good quality, with a low amount of free fatty acids (1.8%) and a concurrently high amount of triacylglycerols (98%). It is a rich source of petroselinic acid (C18:1n-12), an important renewable building block, making up 73% of all fatty acids, with also significant amounts of linoleic acid (14%), oleic acid (6%), and palmitic acid (3%). The oil was characterized by a high unsaponifiable fraction, comprising a substantial amount of phytosterols (6.70 g/kg). The main sterol markers were β-sitosterol (35% of total sterols), stigmasterol (24%), and Δ^7^-stigmastenol (18%). Squalene was detected at an amount of 0.2 g/kg. A considerable amount of tocols were identified (500 mg/kg) and consisted mainly of tocotrienols, with γ-tocotrienol as the major compound. The phospholipid content was low at 0.3%, of which the main phospholipid classes were phosphatidic acid (33%), phosphatidylcholine (25%), phosphatidylinositol (17%), and phosphatidylethanolamine (17%). About 50% of all phospholipids were non-hydratable. The β-carotene content was low at 10 mg/kg, while a significant amount of chlorophyll was detected at about 11 mg/kg. An iron content of 1.4 mg/kg was determined through element analysis of the vegetable oil. The influence of fruit origin on the vegetable oil composition was shown to be very important, particularly in terms of the phospholipids, sterols, and tocols composition.

## 1. Introduction

*Coriandrum sativum* L. is an annual herb from the Apiaceae (Umbelliferae) family that originates from the Near East and Mediterranean area. Currently, India is its main producer with an annual production of around 500,000 tonnes [1]. It is mainly applied as a spice but has also found applications in perfumery and cosmetics, owing to its spicy citrus flavor. Next to this, coriander is well known as a medicinal herb and has been shown to exhibit antioxidant [2], antimicrobial [3,4], anti-inflammatory [5], anxiolytic [6], antidepressant [7], and hypoglycemic activities [8].

Coriander fruits are marked by the presence of two distinct oil fractions, i.e., a vegetable oil and an essential oil fraction, the former being extractable continuously by mechanical pressing of the fruits using twin-screw extrusion technology [9]. While the vegetable oil content of the fruits is typically between 20% and 28%, the essential oil fraction is much smaller with contents of 0.3% to 0.9% [10]. Both the vegetable and the essential oil are characterized by the presence of a key compound, i.e., petroselinic acid and linalool, respectively, representing about 70% of the respective oil fraction. Petroselinic acid is particularly interesting as it constitutes a rather uncommon fatty acid mainly found in the seeds from Apiaceae crops. It represents a positional isomer of oleic acid with its double bond situated at the 6-position rather than the 9-position, which makes it an interesting building block for the chemical industry [11].

Coriander vegetable oil has recently been labelled as a Novel Food Ingredient (NFI) and has been proven safe for consumption as a food supplement by healthy adults with a maximum of 600 mg/day [12]. Next to this, the presence of petroselinic acid gives rise to a wide array of opportunities for coriander vegetable oil. The fatty acid has been shown to exhibit some attractive properties such as anti-aging and anti-inflammatory activity, leading to its application in the cosmetic and functional food industry [13,14]. Further, petroselinic acid displays significant potential for the chemical industry. Oxidative cleavage leads to industrially interesting compounds, as this renders both lauric acid, a commercial surfactant; and adipic acid, a precursor for Nylon 66 [15]. As this position of the double bond is a rather rare feature amongst octadecenoic acids, derivatization may result in the synthesis of some unique and potentially interesting oleochemicals. A recent study dealing with the use of petroselinic acid for the fermentation of novel sophorolipids has shown that petroselinic acid can be isolated from French coriander oil in a high yield of 80% through alkaline hydrolysis of the vegetable oil and subsequent crystallization in absolute ethanol [11]. Baird and Preskett describe the processing of Apiaceae seeds to obtain a solid component rich in petroselinic acid through extraction and crystallization [16].

Most analytical studies have focused on the essential oil part of coriander fruits, while some studies were conducted on vegetable oil extracted from Tunisian, German, and Canadian coriander fruits [17,18,19]. This study aims to provide a full characterization of the vegetable oil extracted from coriander fruits of French origin, which, to the best knowledge of the authors, has not been the subject of any previous studies. Further, determination of the element content of coriander vegetable oil has never been reported. The importance of these results lies within the industrial potential of coriander vegetable oil, as its lipid profile and minor compounds greatly contribute to its added value for several industries.

## 2. Results and Discussion

### 2.1. Vegetable Oil Yield

*n*-Hexane solvent extraction of coriander fruits of French origin yielded a vegetable oil with 22.9% ± 1.0% on a dry weight basis. This is in accordance with the oil yield range of 20%–28% mentioned by Sahib et al. for coriander fruits [10]. Sriti et al. obtained an oil yield of 22% and 16% for Tunisian and Canadian coriander fruits, respectively [18] A different study reported oil yields ranging between 15% and 26% for Tunisian coriander fruits, depending on the fruit maturity [20]. Vietnamese coriander fruits rendered a 20% oil yield upon *n*-hexane solvent extraction, while this was only 11% for Turkish coriander fruits [21,22]. This confirms the strong dependence of the fruit oil content on fruit origin, variety, maturity, and cultivation conditions.

Several studies applied different extraction methods to obtain the coriander vegetable oil. A 2:1 mixture of chloroform and methanol was used to extract the oil from German coriander fruits, leading to a high oil yield of 28% [19]. Kozłowska et al. compared *n*-hexane and 2:1 chloroform/methanol as extracting solvents for Polish coriander oil and found oil yields of 20% and 22%, respectively [23]. British coriander fruits were extracted using the method of Welch, which utilizes 2% sulphuric acid in methanol as a solvent, with a resulting oil yield of 17%–19% [24]. A sustainable process using supercritical CO_2_ extraction and subsequent separation of essential and vegetable oil was applied for Canadian coriander fruits by Mhemdi et al. resulting in an oil yield of 19%, representing 90% of the yield obtained through *n*-hexane solvent extraction [25]. Mechanical pressing has been applied for the extraction of coriander oil without the use of hazardous solvents. For Tunisian coriander fruits, single-screw and twin-screw extrusion lead to an oil yield representing 65% and 47%, respectively, of that obtained through solvent extraction [26,27]. Twin-screw extrusion of French coriander fruits led to a similar oil recovery of 47% [9]. This illustrates the significantly higher extraction capacity of solvent extraction as opposed to mechanical pressing.

### 2.2. Vegetable Oil Composition

The coriander vegetable oil composition was determined through GC and consists of 97.83% ± 1.06% of triglycerides (TAG), 1.05% ± 0.09% of diglycerides (DAG), 0.09% ± 0.02% of monoglycerides (MAG) and 1.03% ± 0.03% of FFA, expressed as petroselinic acid The FFA content was also determined through titration, which led to a higher value (1.80%). This difference may be explained by the fact that through titration, all acidic compounds are neutralized by NaOH. These compounds comprise free fatty acids but also other components that show an acidic character, such as phospholipids. The high amount of TAG and concurrent low FFA content demonstrates that little hydrolysis or enzymatic degradation has occurred in the oil. Similar results were reported by Sriti et al. and Ramadan and Mörsel for Tunisian and German coriander oil, respectively, although in the latter only 91% of TAG and 6% of FFA were detected [17,19]. It is important to highlight that the FFA content is indicative of the oil quality.

### 2.3. Fatty Acid Profile

The distribution and identification of fatty acids in the vegetable oil (Table 1) was determined through gas chromatography of their methyl ester form. From this, it is clear that petroselinic acid (C18:1n-12) is the major fatty acid in coriander vegetable oil, constituting 72.6% of all fatty acids. Other important fatty acids are linoleic acid (C18:2) and oleic acid (C18:1n-9), representing about 14% and 6% of all fatty acids, respectively. Other vegetable oils with similar fatty acid compositions include parsley and celery oil, both rich in petroselinic acid (75% and 64%, respectively) and originating from the Apiaceae family [28]. Further, coriander oil is often compared to high-oleic sunflower oil, which shows a similar composition but has oleic acid, a positional isomer of petroselinic acid, as a major compound (70%–87%) [29].

The results obtained for French coriander oil are similar to the fatty acid profiles found in literature for coriander oil, for which the amount of petroselinic acid ranges from 52% and 66% for Italian and German coriander oil, respectively, to 79% for Vietnamese coriander oil [21,30,31]. Intermediate values of about 72%–76% were reported for Canadian, Tunisian, Polish, British, Indian, and Turkish coriander oil [18,22,23,24,32]. This leads to the conclusion that the fatty acid profile of coriander vegetable oil is not highly dependent on the geographical location [10].

### 2.4. Sterol Composition

**Figure 1:**
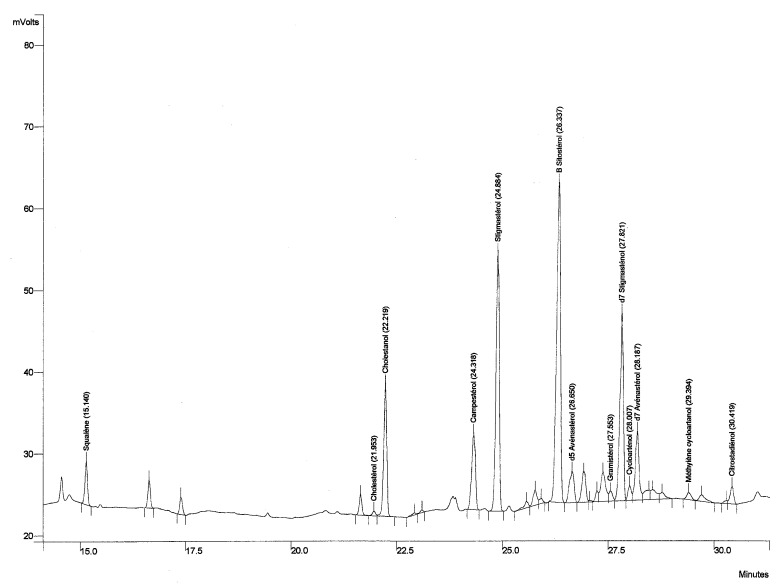
Gas chromatogram from sterol analysis of coriander vegetable oil.

The sterol composition of coriander vegetable oil of French origin is presented in Table 2. The oil is a rich source of phytosterols, comprising a total amount of about 6.70 g/kg. Most vegetable oils show lower sterol contents of between 1 and 5 g/kg, e.g., olive oil with a sterol content of 1.2–2.8 g/kg [33,34]. Edible oils that are rich in phytosterols include rapeseed oil (7.5 g/kg) and corn oil (14 g/kg) [33]. Phytosterols are of importance for the pharmaceutical industry as they are applied as raw materials for steroid production. Next to this, they have been added to margarines and table spreads due to their cholesterol-lowering capacity [35]. The most important compound was found to be β-sitosterol, constituting about 35% of all sterols. Other major compounds include stigmasterol, Δ^7^-stigmastenol, and campesterol at 24%, 18%, and 8% of total sterols, respectively. Besides this, squalene, a compound of interest for the cosmetic industry, was detected in the unsaponifiable matter at an amount of 0.19 ± 0.01 g/kg oil. Most vegetable oils contain 0.05–0.50 g/kg squalene, while rich vegetable sources include olive oil (1.6 g/kg) and amaranthus oil (70 g/kg) [33]. Figure 1 presents the obtained chromatogram from sterol analysis. Sunflower oil shows a similar sterol composition, with β-sitosterol as the main compound representing a more important fraction of 42%–70%, while its total sterol content is significantly lower at 2–5 g/kg [29].

A similar composition was reported by Sriti et al. for Tunisian and Canadian coriander oil, although significant amounts of Δ^5,24^-stigmastadienol were detected, while this was not detected for French coriander oil [17,18,36]. In German, Hungarian, and Polish coriander oil, stigmasterol and β-sitosterol were found in equal amounts, both representing 25%–30% of all sterols, while no Δ^7^-stigmastenol was detected and Δ^5^-avenasterol was more important at about 25% for German and Hungarian coriander oil and 15% for Polish coriander oil [19,23,31]. Reported total sterol contents were always significantly lower than those determined for coriander oil of French origin. Hungarian, Tunisian, and Canadian coriander oil contained between 6.0 and 6.3 g/kg sterols, while this was 5.2 g/kg for German coriander oil and only 3.5 g/kg for Polish coriander oil [18,19,23,31,36]. From this, it is clear that the sterol composition of coriander vegetable oil is strongly dependent on the fruit origin. This has also been reported for other important vegetable oils, such as olive oil, where the sterol content and composition was found to be highly dependent on olive cultivar, fruit ripening, and agro-environmental conditions [37,38].

### 2.5. Tocols Composition

The composition of tocopherols and tocotrienols was measured using HPLC and is listed in Table 3. The total amount of tocols is about 500 mg/kg, which is an intermediate value as vegetable oils usually contain between 200 and 800 mg/kg tocols [33], and will protect the oil from oxidative reactions. The most prominent compound is γ-tocotrienol, representing 71% of all tocols. Other important compounds are α-tocotrienol and δ-tocotrienol at 20% and 5% of all tocols, respectively. Tocotrienols constitute about 95% of all tocols, which is interesting as these compounds have shown great antioxidant capacity and biological activities when compared to tocopherols [39,40].

This composition resembles that of palm oil (Table 3), while sunflower oil is rich in α-tocopherol (400–1100 mg/kg) and typically contains only traces of tocotrienols [29]. The results are consistent with results reported by Matthaus et al. for Vietnamese coriander oil [21]. Tunisian and Canadian coriander oil were shown to exhibit a similar tocol composition, although a significantly lower total amount of tocols was found at about 330 and 210 mg/kg, respectively [17,18]. Coriander fruits of Belgian origin were reported to contain a very low amount of tocols (53 mg/kg) with δ-tocopherol as the main compound and only 6 mg/kg of tocotrienols [41]. Some studies only reported data on the tocopherol fraction. For coriander oil from the USA, Moser and Vaughn found a total amount of 230 mg/kg tocopherols with a high level of α-tocopherol (196 mg/kg) [42]. Hungarian coriander oil showed a very high level of tocopherols (1270 mg/kg) with β-tocopherol as the major compound, making up 50% of all tocopherols, while no β-tocopherol was detected for French coriander oil [31]. The tocols composition of vegetable oils is strongly affected by fruit origin and cultivation conditions, as well as the oil extraction method [43].

### 2.6. Phospholipid Composition

The phospholipid composition of coriander vegetable oil was determined using ^31^P-NMR and is presented in Table 4. The total phospholipid content resulting from this analysis amounts to 0.31%, of which the major phospholipid subclass was found to be phosphatidic acid (PA), constituting 33% of all phospholipids. Other important classes are phosphatidylcholine (PC) at 25% and phosphatidylinositol (PI) and phosphatidylethanolamine (PE), both representing about 17% of all phospholipids. The low phospholipid content of French coriander oil may present advantages during further refining, in particular degumming. Many commercially important crude edible oils are rich in phospholipids, e.g., soybean oil (3.2%), rapeseed oil (2.5%), and sunflower oil (1.5%), and need further refining [33].

In literature, the total amount of phospholipids in coriander oil ranges widely. It varied from 1.5% to 2.0% for German and Tunisian coriander oil, Hungarian coriander oil contained about 0.9% of phospholipids and Prasad et al. reported a very low total amount of phospholipids (0.1%) for Indian coriander oil [19,31,32,44]. For all these oils, a similar composition was reported with PC as the major compound (36%–45%) and significant amounts of PE (25%–32%) and PI (10%–13%) [19,32,44]. The other interesting fact is related to the presence of PA as the major phospholipid in French coriander oil, since in coriander oil from German, Tunisian, or Indian origin, PA was either not detected [19] or detected in amounts of less than 10% [17,32,44].

### 2.7. Pigments Content

Both the β-carotene and chlorophyll content of French coriander vegetable oil was measured using UV spectrophotometry. The β‑carotene content is low at 10.1 ± 0.9 mg/kg compared to palm oil displaying very high β-carotene contents of 500 to 1500 mg/kg [45]. Ramadan and Mörsel have reported a very high β-carotene content of 890 mg/kg for Hungarian coriander vegetable oil [31]. The amount of chlorophyll was found to be relatively high at 11.1 mg/kg. Canola oil is known as the commercial vegetable oil with the highest chlorophyll contents and contains at most 30 mg/kg of chlorophyll [46]. High chlorophyll contents may impart undesirable oil color and render the oil more susceptible to oxidation [46]. As a comparison, sunflower oil contains very low amounts of pigments, showing carotenoid and chlorophyll contents of only 1–1.5 and 0.2–0.5 mg/kg, respectively [29]. To the best knowledge of the authors, no previous studies have reported the chlorophyll content of coriander oil.

### 2.8. Elements Content

The levels of various elements in coriander vegetable oil were determined using ICP analysis and resulted in the values presented in Table 5. The iron content is acceptable at 1.4 mg/kg as the standard for crude vegetable oils is set at 5.0 mg/kg [47]. As an example, crude rapeseed oil shows an iron content of 3.5 mg/kg [48]. These metal traces present an important factor for the oxidative stability of the oil. The phosphorus level of 230 mg/kg is rather low as most crude vegetable oils display phosphorus contents between 200 and 800 mg/kg, while crude rapeseed oil shows a high phosphorus content of 1190 mg/kg [48,49]. The non-hydratable amount of phospholipids may be determined as the sum of the calcium and magnesium content and constitutes about 50% of all phosphorous. This may present a problem for further degumming as the removal of non-hydratable phospholipids requires more complex processes with the use of phosphoric or citric acid [50]. However, recent developments of enzymatic degumming have resulted in an environmentally friendly alternative that reduces the use of chemicals [51].

The total amount of 0.31% of phospholipids, determined through ^31^P-NMR, corresponds to about 131 mg/kg of phosphorous coming from phospholipids (Table 5). This was determined through calculation of the average P/PL factor (0.042) for coriander oil. Of this amount, a little more than half comes from hydratable phospholipids (HPL) such as PC, while the rest comes from non-hydratable phospholipids (NHPL) such as PA. Taking into account the phosphorous content of 230 mg/kg detected by ICP, about 100 mg/kg of phosphorous contained within the vegetable oil comes from an origin other than phospholipids, e.g., inorganic phosphates, glycophospholipids or phytic acid, an important storage compound for phosphorous in seeds.

## 3. Materials and Methods

### 3.1. Material

Coriander fruits were supplied by GSN Semences (Le Houga, France). They consisted of the GSN maintenaire variety and were cultivated in the southwestern part of France. The fruit moisture content was determined gravimetrically according to ISO 665:2000 and was 9.77% ± 0.10% [52].

### 3.2. Chemicals and Reagents

Tocopherol standards were obtained from Calbiochem (San Diego, CA, USA). β-carotene and chlorophyll standards were procured from Fluka (Buchs, Switzerland). Sterol standards, tricaprin, glycerin, monoolein, diolein, triolein, butanetriol, and betulin were purchased from Sigma-Aldrich (St. Louis, MO, USA) and Acros Organics (Morris Plains, NJ, USA). All reagents used in this study were purchased from Sigma-Aldrich, Merck (Darmstadt, Germany), Chem-Lab (Zedelgem, Belgium), or Fisher Scientific (Hampton, NH, USA) and were either analytical or HPLC grade.

### 3.3. Lipid Extraction

Milling of the fruits was executed with a Retsch (Haan, Germany) miller, model ZM100, at 18,000 rpm. For further milling of the cake between extraction steps, a 0.75 mm sieve was applied. Oil extraction was carried out according to ISO 659:2009 with a Soxhlet apparatus and *n*-hexane as the extracting solvent [53].

### 3.4. Physicochemical Analyses

The free fatty acid (FFA) content was determined by titration according to AOCS Ca 5a-40 [54]. The elements content was analyzed through inductively coupled plasma (ICP) analysis (iCAP 6000 series, Thermo Scientific, Waltham, MA, USA) according to AOCS Ca 17-01 and Ca 20-99 [55,56]. The β-carotene content was determined by UV spectrophotometry (UV-1800, Shimadzu, Kyoto, Japan), measuring the absorbance of samples diluted with iso-octane at 446.0 nm. The chlorophyll content was determined according to AOCS Cc 13i-96 with a Shimadzu UV-1800 spectrophotometer [57].

### 3.5. Gas Chromatography

#### 3.5.1. Vegetable Oil Composition

The vegetable oil composition was measured according to the AOCS method Cd 11b-91 [58]. Analytical conditions: chromatograph, Agilent 7890A GC system (Santa Clara, CA, USA); column, Agilent DB-5HT (5% phenyl-methylpolysiloxane), 15 m × 0.32 mm × 0.10 µm; helium as the carrier gas at a rate of 5 mL/min; cold-on-column injection with an injection volume of 1 µL; column temperature of 50–200 °C (15 °C/min), 200–290 °C (3 °C/min), 290 °C for 10 min, 290–360 °C (10 °C/min), 360 °C for 15 min; flame ionization detector (FID) with a temperature of 380 °C. The fatty compounds were identified using authentic standards (glycerin, oleic acid, monoolein, diolein, and triolein), as described in the method.

#### 3.5.2. Acid Composition

Fatty acid methyl esters were prepared according to the AOCS method Ce 2-66 [59]. Analytical conditions: chromatograph, Agilent 6890N GC System; column, Agilent DB-23 (50% cyanopropyl-methylpolysiloxane), 30 m × 0.25 mm × 0.25 μm; helium as the carrier gas at a rate of 1.0 mL/min; injection temperature of 250 °C; column temperature of 110 °C for 5 min, 110–215 °C (5 °C/min), 215 °C for 24 min; detection temperature of 280 °C. The fatty acid methyl esters were identified by comparison with external standards purchased from Nu Check Inc. (Elysian, Chicago, IL, USA).

#### 3.5.3. Sterol Composition

The method used to analyze the sterol composition was based on the one used by Roche et al. [60]. Exactly 100 µg of cholestanol was added as an internal standard through a 2 mg/mL solution in chloroform. After chloroform evaporation, vegetable oil samples of 100 mg were added. Saponification was carried out through addition of 2 mL of a 1 M potassium hydroxide in ethanol solution. Samples were vortex mixed and heated to 75 °C for 20 min in a water bath. After cooling to ambient temperature, the unsaponifiable matter was extracted through the addition of 1 mL of distilled water and 6 mL of cyclohexane and vortex mixing. After separation of the layers, the organic phase was collected. 160 µL of the extract was silylated by adding 40 µL of a 1% TMCS in BSTFA solution. Samples were subjected to GC analysis.

Analytical conditions: chromatograph, Perkin Elmer AutoSystem GC system (Waltham, MA, USA); column, Varian CPSil 8CB (5% phenyl-dimethylpolysiloxane), 30 m × 0.25 mm × 0.25 µm; helium as the carrier gas with a column head pressure of 100 kPa; on-column injection with an injection volume of 1 µL; injection temperature of 55 °C for 0.5 min, 55–360 °C (200 °C/min), 360 °C for 30 min; column temperature of 160 °C for 0.5 min, 160–260 °C (20 °C/min), 260 °C for 5.5 min, 260–300 °C (2 °C/min), 300 °C for 10 min, 300–360 °C (45 °C/min), 360 °C for 3 min; detection temperature of 360 °C.

### 3.6. High-Performance Liquid Chromatography

Tocopherols and tocotrienols were analyzed through HPLC according to AOCS Ce 8-89 [61]. Analytical conditions: chromatograph, Agilent 1260 Infinity HPLC system; pump, G1511C quaternary pump; column, Grace normal phase, 250 mm × 4.6 mm × 5 µm; detector, HP series 110 fluorescence detector with an extinction of 290 nm and an emission of 330 nm; *n*-hexane/2-propanol (99.5/0.5 *v*/*v*) as the mobile phase at a rate of 1 mL/min at isocratic conditions; injection volume of 20 µL; column temperature of 25 °C; runtime of 30 min. The tocotrienol isomer contents were calculated based on the standard peak areas of their related tocopherol analogs.

### 3.7. Nuclear Magnetic Resonance

Determination and identification of the phospholipid content was carried out according to the ^31^P-NMR quantification method from Spectral Service GmbH (Köln, Germany) [62]. NMR analyses were performed on a Bruker Avance III Nanobay 400 MHz NMR spectrometer (Bruker, Billerica, MA, USA) with a 5 mm BBFO Z-gradient high-resolution probe. ^31^P-NMR spectra were recorded at 162 MHz. Results were processed using Topspin software (Topspin 3.5, Bruker).

Vegetable oil samples of between 500 and 600 mg were weighed. 5 mg of triphenyl phosphate was added as an internal standard. 1.5 mL of a 1/1/1 solvent mixture of CDCl_3_, methanol and a 0.2 M Cs-EDTA solution was added. Samples were stirred for 5 min and centrifuged at 4000 rpm for 5 min for the separation of the layers. 0.5 mL of the lower organic phase was collected and subjected to ^31^P-NMR analysis.

All analyses were carried out in triplicate and results are presented as the mean ± the standard deviation (SD).

## 4. Conclusions

*Coriandrum sativum* L. represents an interesting novel renewable resource, exhibiting a wide range of biological activities and containing both an essential oil and a vegetable oil fraction. This study comprises a full characterization of the vegetable oil fraction of French coriander fruits. This oil was shown to be of good quality and rich in petroselinic acid, an uncommon positional isomer of oleic acid and important renewable building block. Further, the oil contains a fair amount of tocols, of which 95% are tocotrienols, exhibiting strong antioxidant capacity, with γ-tocotrienol as the major compound. Next to this, French coriander oil was found to be particularly rich in phytosterols, industrially important compounds with significant human health benefits, with an amount of 6.70 g/kg and β-sitosterol as the main sterol marker. As part of the unsaponifiable matter, squalene was detected at 0.2 g/kg. Further, significant differences between coriander fruit origins were detected in the phospholipids, where the French coriander oil showed a substantially lower total amount of only 0.3% with phosphatidic acid as the major compound. A low β-carotene content (10 mg/kg) and a significant amount of chlorophyll were detected. The amount of iron in the oil was acceptable at 1.4 mg/kg. This study has shown that the coriander fruit origin has a significant influence on the composition of the vegetable oil, with phospholipids, phytosterols, tocols, and pigments as the main compounds being affected. It was further shown that the French variety may have high industrial potential through the presence of significant amounts of phytosterols and tocotrienols and a high petroselinic acid content.

## Figures and Tables

**Table 1:** Fatty acid profile of coriander vegetable oil.

Fatty Acid	Content (%)
C6:0	0.1 ± 0.1
C16:0	2.9 ± 0.1
C16:1	0.4 ± 0.1
C16:1t	0.2 ± 0.1
C16:1c	0.2 ± 0.1
C17:0	<0.1
C18:1t	0.7 ± 0.1
C18:0	<0.1
C18:1n-12	72.6 ± 0.4
C18:1n-9	6.0 ± 0.3
C18:1n-7	1.2 ± 0.1
C18:2	13.8 ± 0.3
C18:2t	0.1 ± 0.1
C18:2c	13.7 ± 0.3
C18:3	0.2 ± 0.1
C18:3t	0.1 ± 0.1
C18:3c	0.1 ± 0.1
C20:0	0.1 ± 0.1
C20:1	0.2 ± 0.1
SFA	3.2 ± 0.1
MUFA	81.2 ± 0.4
PUFA	14.0 ± 0.3
Identified	98.3 ± 0.2

SFA: saturated fatty acids; MUFA: monounsaturated fatty acids; PUFA: polyunsaturated fatty acids.

**Table 2:** Sterol composition of coriander vegetable oil.

Sterol	Content (g/kg)
Cholesterol	0.02 ± 0.01
Campesterol	0.54 ± 0.01
Stigmasterol	1.61 ± 0.02
β-Sitosterol	2.31 ± 0.03
Δ^5^-Avenasterol	0.27 ± 0.01
Δ^7^-Stigmastenol	1.22 ± 0.03
Δ^7^-Avenasterol	0.40 ± 0.01
Gramisterol	0.07 ± 0.01
Citrostadienol	0.10 ± 0.01
Cycloartenol	0.08 ± 0.01
Methylene cycloartanol	0.06 ± 0.01
Total sterols	6.68 ± 0.02

**Table 3:** Tocol composition of coriander and palm oil (mg/kg).

Tocol	Coriander Oil	Palm Oil
α-tocopherol	12.4 ± 0.1	147.5
α-tocotrienol	98.0 ± 2.0	146.8
β-tocopherol	n.d.	n.d.
γ-tocopherol	10.1 ± 0.2	19.5
γ-tocotrienol	350.3 ± 6.7	283.0
δ-tocopherol	n.d.	n.d.
δ-tocotrienol	25.7 ± 0.3	49.7
Total tocols	496.5 ± 8.3	646.5

n.d.: not detected.

**Table 4:** Phospholipid composition of coriander vegetable oil.

Phospholipid Subclass	% by Weight
Phosphatidic acid	32.5 ± 1.0
Phosphatidylcholine	25.4 ± 2.9
Phosphatidylinositol	17.0 ± 4.7
Phosphatidylethanolamine	16.7 ± 2.7
Phosphatidyl glycerol	8.1 ± 0.5
1-lysophosphatidylcholine	0.5 ± 0.3

**Table 5:** Elements content of coriander vegetable oil (mg/kg).

Ca	Fe	K	Mg	Na	P	P from PL (NMR)	HPL	NHPL	P Different Origin
92.8 ± 0.8	1.4 ± 0.1	73.1 ± 0.5	35.8 ± 0.2	5.2 ± 0.1	230.7 ± 0.4	131.2 ± 8.9	75.2 ± 7.8	56.4 ± 3.1	99.5 ± 8.9

HPL: hydratable phospholipids; NHPL: non-hydratable phospholipids.
